# Study on association of serum uric acid levels with bipolar disorder: systematic review and meta-analysis in Chinese patients

**DOI:** 10.1186/s12991-023-00450-5

**Published:** 2023-05-18

**Authors:** Haihan Chen, Fengli Sun, Weidong Jin

**Affiliations:** 1 Department of Psychiatry, Second Clinical College, Zhejiang Chinese Medical University, Hangzhou, China; 2 Department of Psychiatry, Zhejiang Province Mental Health Center, Zhejiang Province Tongde Hospital, Hangzhou, China

**Keywords:** Bipolar disorder, Mania, Uric acid, Meta-analysis

## Abstract

**Background:**

The purine system represented by uric acid may be involved in the pathogenesis of bipolar disorder, This study intends to explore the association of serum uric acid levels with bipolar disorder in Chinese patients through meta-analysis.

**Methods:**

Electronic databases, including PubMed, Embase, Web of Science, and China National Knowledge Infrastructure (CNKI), searching from inception to December 2022. Randomized Controlled Trials that reported serum uric acid levels and bipolar disorder were included. Two investigators independently extracted data and RevMan5.4 and Stata14.2 were used for statistical analyses.

**Results:**

Twenty-eight studies with 4482 bipolar disorder, 1568 depression, 785 schizophrenia, and 2876 healthy control subjects were included in this meta-analysis. The results of the meta-analysis showed that serum uric acid levels in the bipolar disorder group were significantly higher than those in depression [SMD 0.53 (0.37, 0.70), *p* < 0.00001], schizophrenia [SMD 0.27 (0.05, 0.49), *p* = 0.02] and healthy control group [SMD 0.87 (0.67, 1.06), *p* < 0.00001]. Subgroup-analysis showed that in Chinese people with bipolar disorder, uric acid levels of the manic episode were higher than the depressed episode [SMD 0.31 (0.22, 0.41), *p* < 0.00001].

**Conclusion:**

Our results indicated a strong association between serum uric acid levels and bipolar disorder in Chinese patients, but further studies about whether uric acid levels can be a biomarker for bipolar disorder still need to investigate.

## Introduction

Bipolar disorder (BD) is a severe psychiatric disorder, characterized by manic, depressive, and mixed episodes [1]. Onset typically occurs between adolescence and early adulthood [2], and it is liable to cause a decline in cognitive function and may be accompanied by mental symptoms, resulting in the impaired social function of patients [3]. The World Mental Health Survey Initiative reported lifetime and 12-month prevalence estimates for bipolar disorders of 2.4% and 1.5% respectively [4]. In addition, bipolar disorder is a psychiatric disease with the highest suicide rate, among which up to mostly self-inflicted suicide occurs in the stage of the bipolar depressive episode. Patients with depressive disorder and bipolar depression are often at risk of turning to mania in clinical practice, and some patients have been diagnosed for many years and delaying the correct treatment because of depression as the first symptom.

The exact pathophysiological pathogenesis of bipolar disorder is still unclear, and it is generally believed that genetic factors are the main cause of the disease, while acquired environmental factors promote the disease [5]. Some studies have shown that the purinergic system with uric acid is closely related to the occurrence and development of bipolar disorder [6]. As one of the non-enzymatic antioxidant systems, uric acid may participate in the pathogenesis of bipolar disorder through oxidative stress and other mechanisms. The latest findings of Oliveira [7] suggest that serum UA levels demonstrated a very good‐to‐excellent prognostic accuracy as a biomarker for conversion to BD in depressed subjects, so it is crucial to explore the association between uric acid levels and bipolar disorder.

This study intends to conduct a meta-analysis of serum uric acid levels in bipolar disorder, depression, schizophrenia, and healthy people, comparing the differences in uric acid levels among different subtypes of bipolar disorder, to provide reference and evidence-based medical support for clinical diagnosis and treatment.

## Materials and methods

### Search strategy

Electronic databases, including PubMed, Embase, Web of Science, and China National Knowledge Infrastructure (CNKI), were searched from inception to December 2022. To find relevant original articles, we used the following terms: “uric acid”, “bipolar disorder”, “hyperuricemia”, “hyperuricaemia”, “depressive disorder”, “depression”, “mania” and “schizophrenia”. Two investigators independently screened the titles and abstracts of the studies according to the eligibility criteria, the full texts were reviewed for further selection, and in case of disagreement a third expert will be consulted.

### Inclusion and exclusion standard

The inclusion criteria were as follows: (1) published studies about the association of serum uric acid levels with bipolar disorder in Chinese patients, unlimited gender; (2) the diagnosis of bipolar disorder, depression, and schizophrenia were based on DSM-4/5 and ICD-10; (3) all studies with full texts, can be used for data extraction. The exclusion criteria were as follows: (1) reviews, systematic evaluations, or animal experiments; (2) duplicate literature and non-peer-reviewed material; (3) data were incomplete or unable to extract serum uric acid values.

### Data extraction

The main characteristics extracted were as follows: first author, the year of publication, study group, sample size, the serum uric acid levels (mean ± SD) in the bipolar disorder and control groups, and the score of the Newcastle–Ottawa Quality Assessment Scale [8]. The NOS scale was used to assess the quality of each study. To integrate more complete and accurate data, one investigator (FLS) contacted the corresponding authors of studies that did not have explicit data or other unpublished studies.

### Statistical analyses

Statistical analyses were conducted by RevMan5.4 and Stata14.2, the standard mean differences (SMDs) of different studies and corresponding 95% confidence intervals (CIs) were used to estimate the association between the experimental group and control group. Heterogeneity among studies were assessed using the I^2^ statistic, with values of 25% (low), 50% (moderate), and 75% (high) [9]. All studies about differences in uric acid levels were estimated according to the following six comparisons: (1) bipolar disorder vs healthy controls; (2) bipolar disorder vs depression; (3) bipolar disorder vs schizophrenia; (4) BD manic episode vs BD depressed episode; (5) BD manic episode vs BD mixed episode; (6) BD depressed episode vs BD mixed episode.

## Results

### Study selection and characteristics

**Fig. 1:**
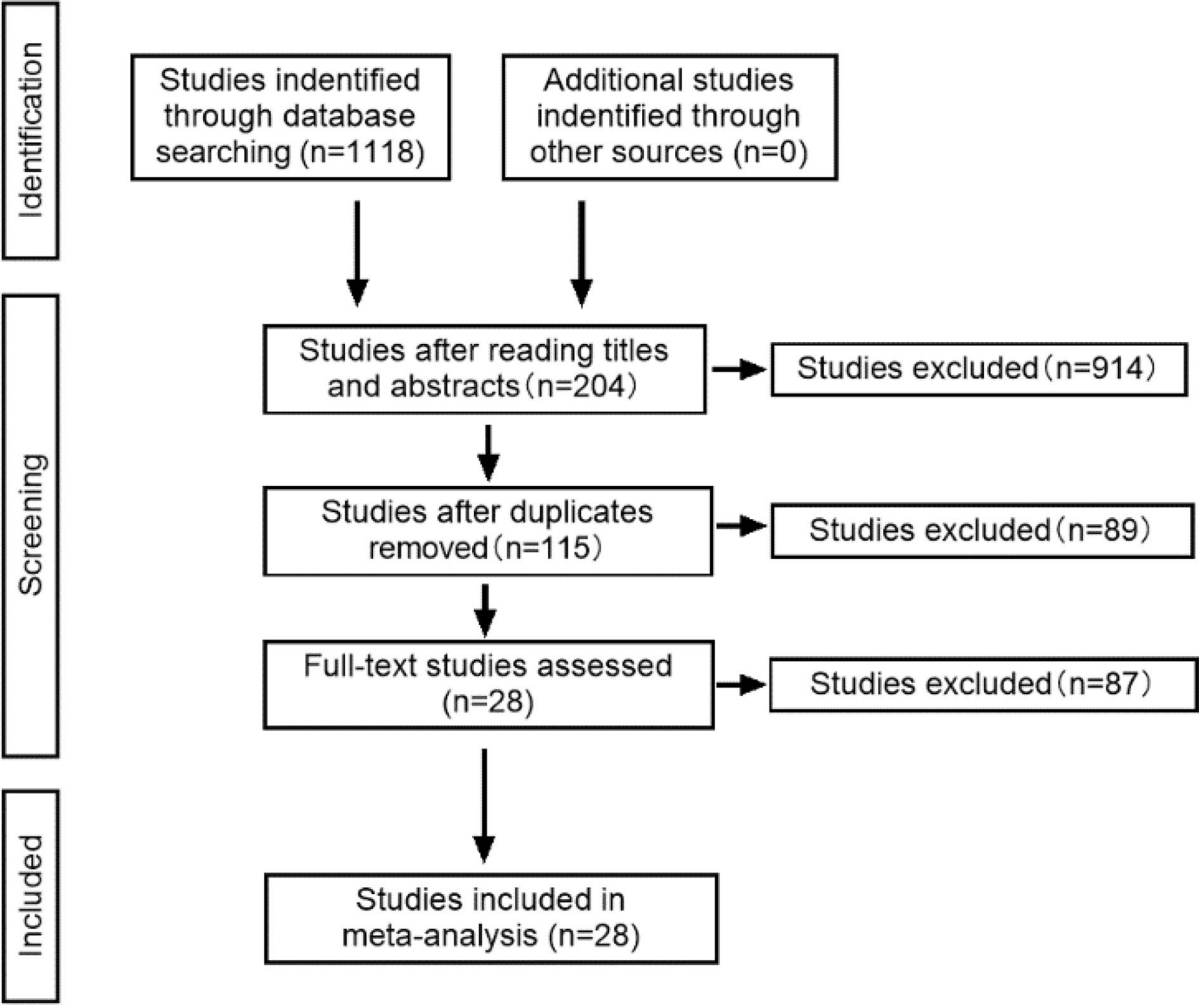
Steps of document retrieval

We retrieved 1118 studies from electronic databases, after reading titles and abstracts through the literature management software, 914 non-conforming studies were excluded, and 176 studies were excluded by full-text reading. Finally, 28 studies were included in the meta-analysis. The steps for document retrieval are shown in Fig. 1.

The characteristics of the twenty-eight included studies are presented in Table 1 [5, 10–36]. The studies were published between 2012 and 2022 and published in Switzerland, Canada, England, and China. A total of 9711 patients with bipolar disorder, depression disorder, schizophrenia, and healthy control were included.

**Table 1:** Description of included studies

Study	Year	Comparison	UA, Mean ± SD (μmol/L)	Sample size	NOS
Wan JL 1	2022	Bipolar Disorder/Healthy Control	284.83 ± 103.35/282.55 ± 57.8	60/60	8
Lin XB	2022	Bipolar Disorder/Healthy Control/BD manic episode/BD depressed episode	358.42 ± 78.96/281.96 ± 72.57/365.84 ± 72.57/349.35 ± 68.74	84/84/51/33	8
Wan JL 2	2022	BD manic episode/BD depressed episode	315.67 ± 92.57/284.83 ± 103.35	55/54	7
Wu Y	2022	Bipolar Disorder/Healthy Control/BD manic episode/BD depressed episode	380.23 ± 105.28/311.65 ± 82.12/395.09 ± 105.51/338.42 ± 93.6	183/135/135/48	6
Li SY	2021	Bipolar Disorder/Healthy Control	463.85 ± 41.48/285.47 ± 53.14	48/48	8
Li ZL	2021	Bipolar Disorder/Healthy Control/BD manic episode/BD depressed episode/BD mixed episode	378.69 ± 76.52/305.76 ± 56.94/378.94 ± 67.89/380.72 ± 68.51/373.25 ± 63.84	132/132/66/39/27	7
Xu RH	2020	Bipolar Disorder/Healthy Control/Schizophrenia/BD manic episode/BD depressed episode	350.28 ± 101.26/280.79 ± 70.64/318.79 ± 84.56/368.55 ± 103.59/321.39 ± 95.64	160/120/120/98/62	8
Dong R	2020	Bipolar Disorder/Healthy Control/Schizophrenia	357.26 ± 80.1/272.81 ± 70.82/318.62 ± 70.37	133/133/133	6
Bai XY	2019	Bipolar Disorder/Healthy Control/BD manic episode/BD depressed episode/BD mixed episode	340 ± 77/268 ± 64/350 ± 87/320 ± 66/355 ± 75	96/144/30/36/30	7
Xing ZQ	2019	Bipolar Disorder/Healthy Control	461.59 ± 60.4/283.13 ± 56.73	104/72	7
Zhang HW	2019	Bipolar Disorder/Healthy Control/BD manic episode/BD depressed episode	358.27 ± 79.41/282.53 ± 73.56/364.74 ± 77.28/349.41 ± 69.87	98/50/60/38	6
Yuan ZW	2019	BD manic episode/BD depressed episode/BD mixed episode	357.75 ± 89.83/331.31 ± 72.4/351.89 ± 50.44	90/67/23	7
Zhang LG	2018	Bipolar Disorder/Healthy Control	371.17 ± 104.63/301.1 ± 78.4	105/105	7
Zhang XM	2018	Bipolar Disorder/Healthy Control/Depression/BD manic episode/BD depressed episode/BD mixed episode	353.46 ± 114.57/296.55 ± 84.03/292.7 ± 85.12/364.02 ± 103.03/330.18 ± 129.83/352.4 ± 138.1	183/130/88/116/52/15	7
Li B	2018	Bipolar Disorder/Healthy Control/BD manic episode/BD depressed episode/BD mixed episode	378.85 ± 76.72/306.95 ± 56.93/375.09 ± 67.71/381.23 ± 68.54/369.37 ± 63.63	92/92/36/45/11	6
Wang Y	2017	Bipolar Disorder/Healthy Control/Schizophrenia/BD manic episode/BD depressed episode	349.44 ± 106.21/281.84 ± 70.93/318.81 ± 85.58/365.54 ± 103.11/321.45 ± 107.68	322/322/77/197/125	7
Xu YX	2016	Bipolar Disorder/Healthy Control/BD manic episode/BD depressed episode	389.43 ± 83.22/282.55 ± 70.24/391.55 ± 84.88/384.95 ± 80.61	109/74/74/35	7
Chen HM	2016	Bipolar Disorder/Healthy Control/Schizophrenia/BD manic episode/BD depressed episode	349.34 ± 107.21/280.94 ± 71.9/319.71 ± 84.48/366.45 ± 104.01322.45 ± 107.69	126/126/69/77/49	8
Chen WX	2021	Bipolar Disorder/Healthy Control/Schizophrenia/BD manic episode/BD depressed episode	346.76 ± 100.94/276.9 ± 50.77/310 ± 76.54/367.9 ± 100.56/319.86 ± 95.28	80/80/50/38/42	5
Deng SS	2019	Bipolar Disorder/Healthy Control	398.09 ± 98.5/354.9 ± 84.87	63/100	5
Li Y	2017	Bipolar Disorder/Healthy Control	369.12 ± 107.49/301.32 ± 76.02	88/88	6
Du HM	2022	Bipolar Disorder/Healthy Control/Depression	349.61 ± 102.02/331.19 ± 104.03/308.7 ± 91.53	86/66/73	7
Li SY	2022	Bipolar Disorder/Healthy Control	377.87 ± 102.15/356.04 ± 100.22	182/182	7
Zhu YC	2022	Bipolar Disorder/Depression	351.4 ± 106.87/311.9 ± 93.58	955/1188	6
Lu Z	2021	Bipolar Disorder/Healthy Control/Depression/BD manic episode/BD depressed episode	354.02 ± 88.75/296.27 ± 68.77/282.13 ± 77.98/367.84 ± 92.92/337.93 ± 81.54	119/180/95/64/55	8
Chen JX	2018	Bipolar Disorder/Healthy Control	348.5 ± 91.8/300.2 ± 76.6	318/160	8
Yang XD	2018	Bipolar Disorder/Healthy Control	354.64 ± 90.37/332.96 ± 88.7	141/151	8
Wen SL	2012	Bipolar Disorder/Healthy Control/Depression/Schizophrenia	323.04 ± 108.7/315.76 ± 87.5/271.97 ± 77.5/341.03 ± 106.84	126/42/124/336	7

### Meta-analysis

#### Bipolar disorder vs healthy control

**Fig. 2:**
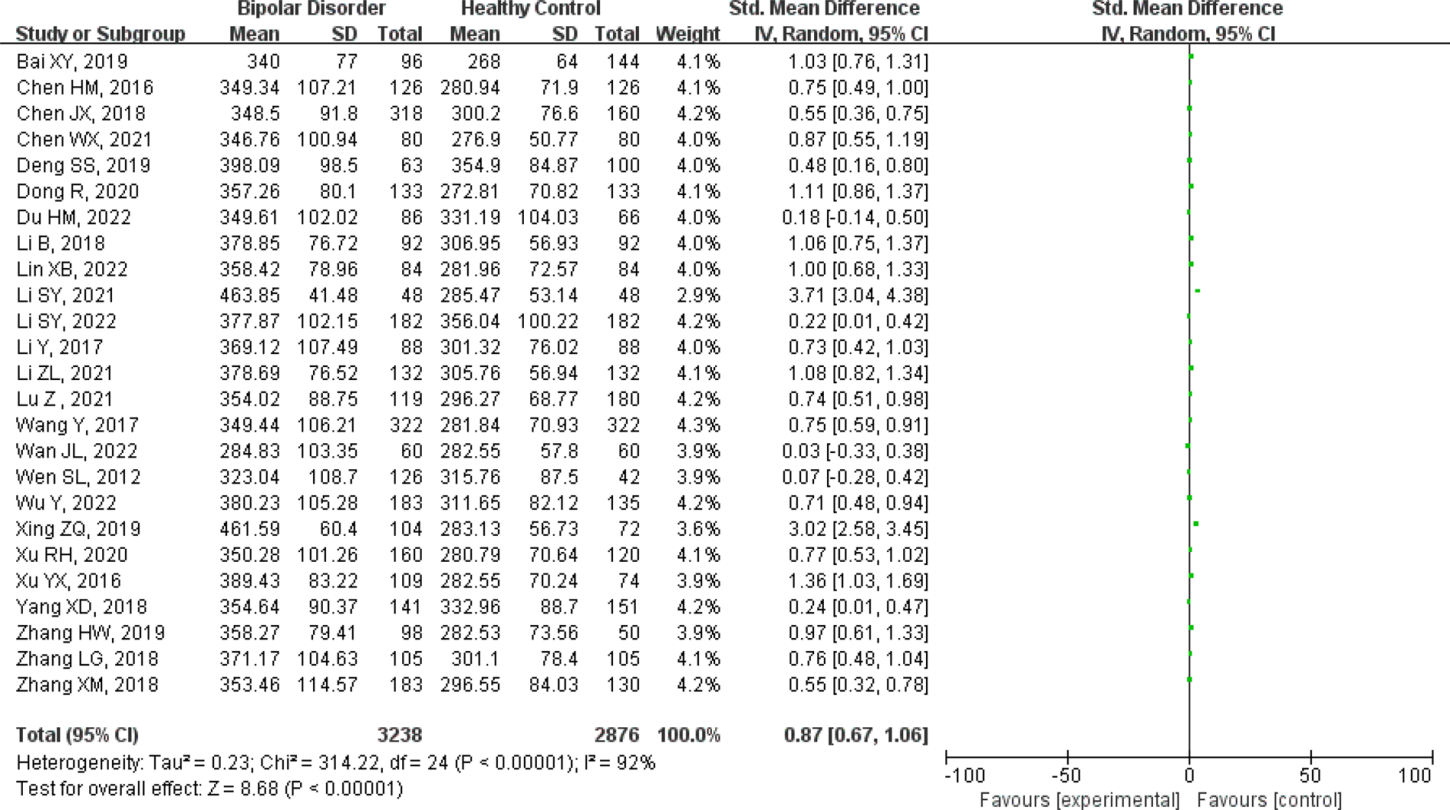
Forest plot for serum uric acid levels in the bipolar disorder compared with the healthy control

**Fig. 3:**
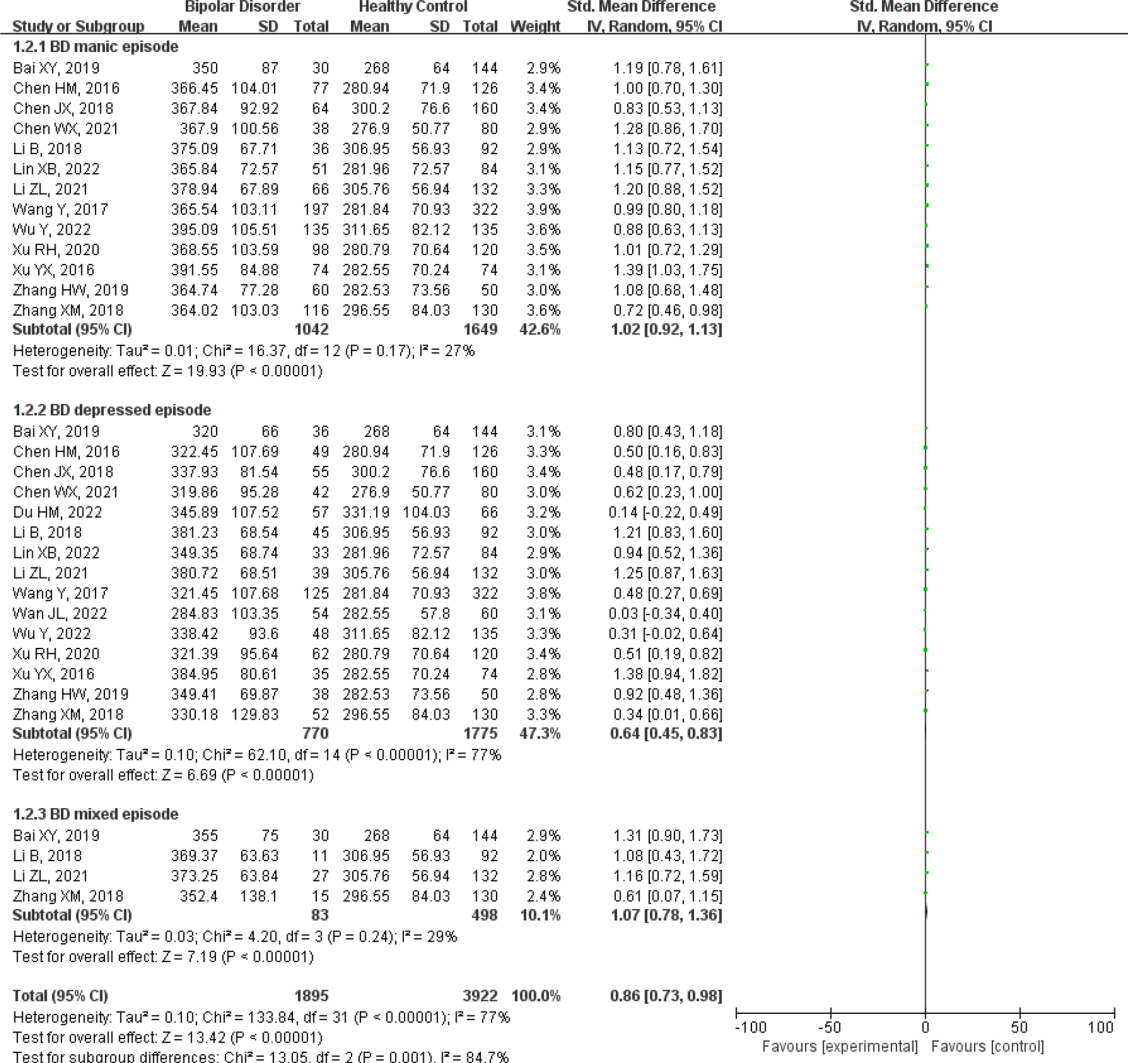
Forest plot for subgroup analysis of serum uric acid levels in the bipolar disorder compared with the healthy control

A total of twenty-five studies were included, including 3238 patients with bipolar disorder and 2876 healthy controls [10–12, 14, 16–19, 21–36]. Figure 2 forest plot for serum uric acid levels in the bipolar disorder group compared with the healthy control group. A random-effects model was used because high levels of heterogeneity were observed among the twenty-five studies. Serum uric acid levels were significantly higher in bipolar disorder patients than healthy controls [SMD 95%CI 0.87 (0.67 ~ 1.06); *Z* = 8.68 (*p* < 0.00001); *I*^2^ = 92%)]. To explore the source of heterogeneity, we conducted a further subgroup analysis of studies that provided data on specific subtypes of bipolar disorder. The forest plot for subgroup analysis is shown in Fig. 3. The results showed that the uric acid levels among different subgroups of bipolar disorder are a major source of high heterogeneity, especially in the group of BD depressed episode, there was low heterogeneity in BD manic/mixed episode groups.

#### Bipolar disorder vs depression

**Fig. 4:**
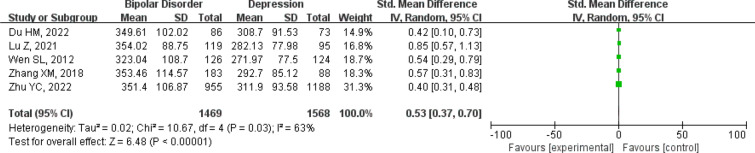
Forest plot for serum uric acid levels in bipolar disorder compared with the depression

When comparing serum uric acid levels in the bipolar disorder group with the depression group, we included 1469 patients with bipolar disorder and 1568 patients with depression [11, 13, 19, 30, 36]. The forest plot for serum uric acid levels in the different groups is shown in Fig. 4. The results showed that serum uric acid levels were significantly higher in bipolar disorder patients than in depression patients [SMD 95%CI 0.53 (0.37 ~ 0.70); *Z*= 6.48 (*p*< 0.00001); *I*^2^ = 63%)]. This comparison also had high heterogeneity, whereas we could not assess publication bias due to the lack of eligible studies.

#### Bipolar disorder vs schizophrenia

**Fig. 5:**
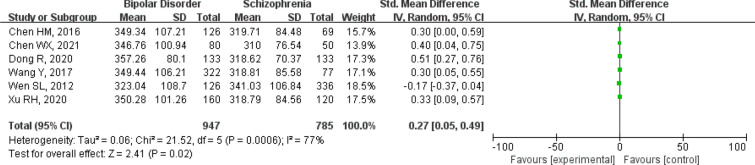
Forest plot for serum uric acid levels in bipolar disorder compared with the schizophrenia

We conducted a meta-analysis of six studies to compare bipolar disorder with schizophrenia, 947 patients with bipolar disorder and 785 patients with schizophrenia were included [18, 21, 22, 33, 35, 36]. Bipolar disorder had increased uric acid levels with an SMD [95%CI 0.27 (0.05 ~ 0.49); *Z* = 2.41 (*p* = 0.02); I^2^ = 77%)]. The heterogeneity was also high, so we chose the random-effects model. The forest plot for serum uric acid levels in the two groups is shown in Fig. 5.

#### BD manic episode vs BD depressed episode

**Fig. 6:**
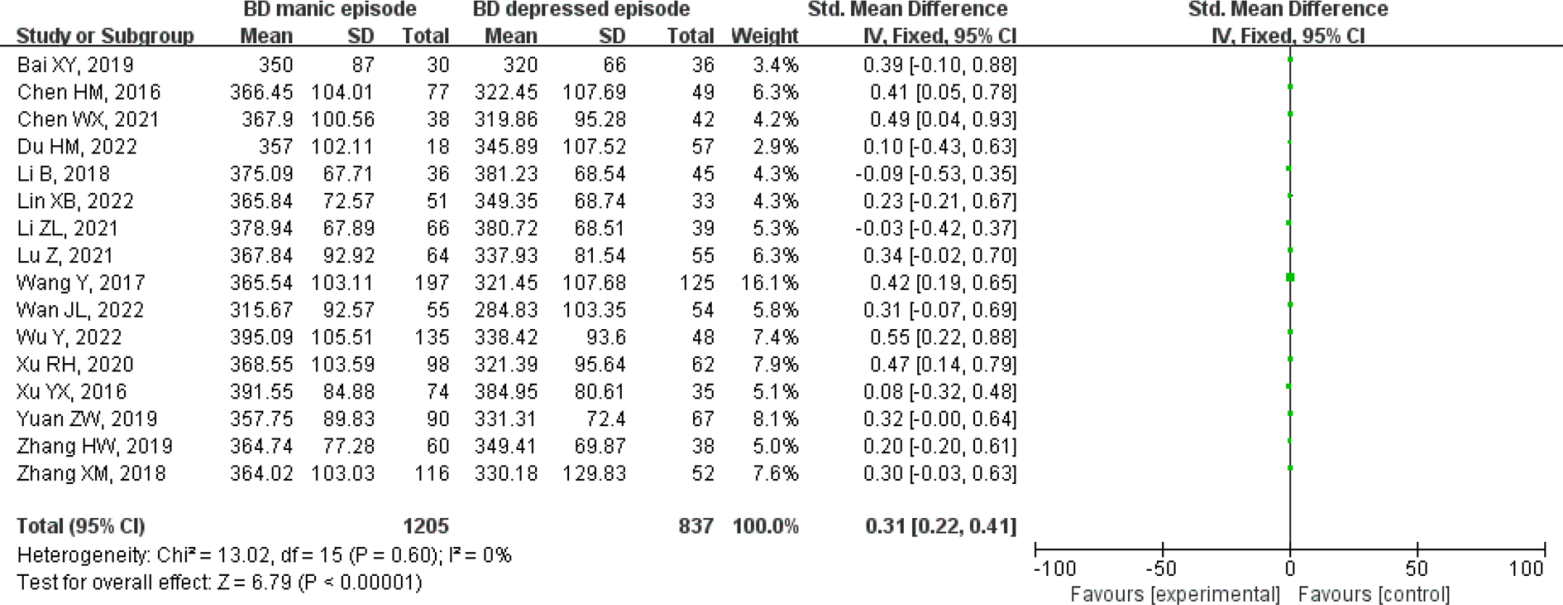
Forest plot for serum uric acid levels in the BD manic episode compared with the BD depressed episode

We compared uric acid levels among different subtypes of bipolar disorder, a total of sixteen studies were compared, including 1205 patients with manic episodes and 837 depressed episodes [5, 11, 14–16, 18–20, 22, 25, 26, 30, 31, 33–35]. Figure 6 shows the serum uric acid levels were significantly higher in manic episode patients than depressed episode patients [SMD 95%CI 0.31 (0.22 ~ 0.41); *Z* = 6.79 (*p* < 0.00001); I^2^ = 0%)], there was low heterogeneity in this comparison, so we chose the fixed-effects model. The results of the comparison showed that uric acid levels may play an important role in distinguishing different subtypes of bipolar disorder.

#### BD mixed episode vs BD manic episode/depressed episode

Finally, we included six studies to analyze the relationship about serum uric acid levels between BD mixed episode and BD manic episode/depressed episode [5, 11, 20, 25, 30, 31]. However, we did not find any significant differences in uric acid levels comparing, respectively, mixed/manic (*p* = 0.63) and mixed/depressed (*p* = 0.16).

#### Sensitivity analysis and quality assessment

**Fig. 7:**
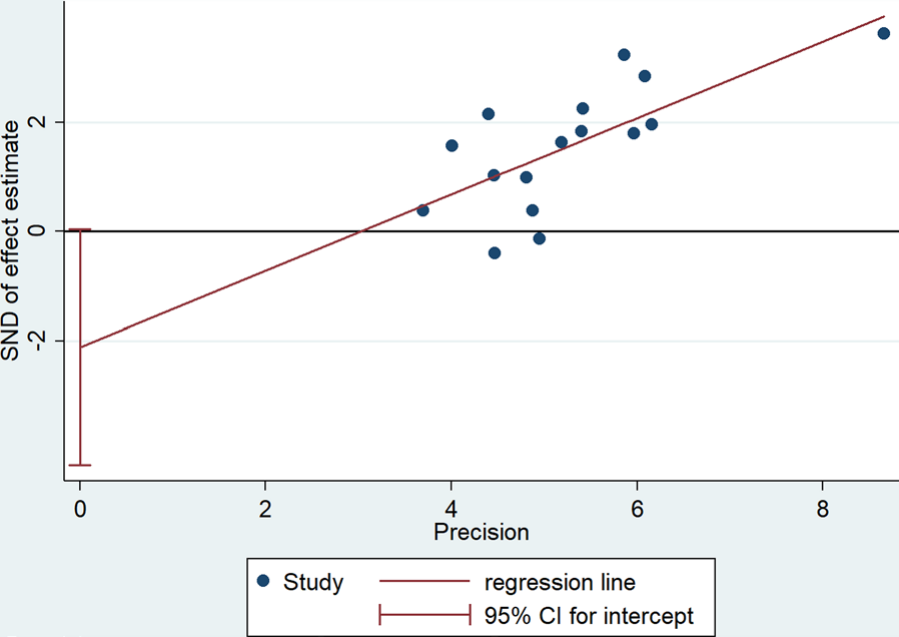
Funnel plot about BD manic episode vs BD depressed episode

A “leave-one-out sensitivity analysis” was performed to evaluate the impact of the heterogeneity between all studies. None of the results about bipolar disorder vs healthy control and the comparison of different subtypes of bipolar disorder were altered after any one study was excluded. However, in the comparison about Bipolar disorder with schizophrenia or depression, the results significantly affected the pooled results (*I*^2^ dropped from > 50 to < 50%) after excluding one study respectively [13, 19, 36]. Therefore, we hypothesized that the study of these three studies may be the source of heterogeneity in this meta-analysis. The risk of publication bias about BD manic episode vs BD depressed episode was analyzed by Egger’s regression test (*p* > 0.05), the results suggested that there was no significant publication bias in the meta-analysis, and the funnel plot is shown in Fig. 7.

## Discussion

Uric acid is the ultimate product of the decomposition in the body’s purine compounds, and UA has a large correlation with oxidative stress. Studies have shown that the purine system may be involved in the regulation of the patient’s cognition, mood, exercise, sleep, and behavior, the effect of purine on neurotransmitter activity probably leads to the emergence of mental diseases [37]. The study of the relationship between purine and BD can go back to the nineteenth century when researchers found that some gout patients had a common emotional disorder, and the symptoms had been reduced by the treatment of lithium [19]. Recently, a long-term epidemiological study by Chung [38] found that the risk of uric acid levels in BD patients was significantly higher than in healthy people, the bipolar disorder and hyperuricemia could be similar neurobiochemical basis between the two diseases. In normal physiological conditions, UA as a non-enzymatic antioxidant has the effect of preventing superoxide dismutase degeneration, and enhances the antioxidant effect of the erythrocyte membrane, by reducing the level of oxidative stress, preventing the apoptosis of the cells, and preventing the oxidative stress damage from further aggravation. However, the high level of UA will be transformed into a powerful oxidant [39], and the high oxidation level will cause the cell membrane to have a chain oxidation reaction, damaging the stability, liquidity, and permeability of the cell membrane, and leading to the development of bipolar disorder [20].

To the best of our knowledge, there is few systematic reviews and meta-analysis to explore the association between serum uric acid levels and bipolar disorder. Our meta-analysis included 28 studies with 4482 bipolar disorder, 1568 depression, 785 schizophrenia, and 2876 healthy control subjects in Chinese patients, showed that serum uric acid levels were significantly higher in bipolar disorder patients than in other control subjects, and in different subtypes of bipolar disorder, serum uric acid levels were significantly higher in the manic episode than in depressed episode. It suggests that the effect of high serum uric acid levels seems to be selective for a manic episode.

The results of the current meta-analysis were in agreement with other studies. For example, one previous study [40] also found that serum uric acid levels were significantly increased in bipolar disorder patients compared with healthy controls. These results indicate that abnormal purine metabolism and oxidative stress play an important role in the pathogenesis of bipolar disorder. Abnormal purine metabolism in bipolar disorder leads to mood disorders by affecting the activity of neurotransmitters such as dopamine, glutamate, GABA, and 5-HT [41, 42]. Some evidence from genetic studies also emphasizes the key role of the purinergic system during manic episodes, elevated uric acid may be a specific phenomenon resulting from metabolic abnormalities during manic episodes [43]. The Bartoli’s study [44] found that although gender, metabolic syndrome and triglycerides have a special effect on uric acid, but after controlling the factor of gender, most of the effects of bipolar disorder on uric acid are direct and only influenced by some metabolic parameters.

Individuals with high uric acid levels are also more likely to exhibit higher drive and hyperactive or irritable temperament [45]. A randomized controlled trial of allopurinol adjuvant therapy in patients with bipolar manic episodes concluded that allopurinol as a xanthine oxidase inhibitor can increase adenosine levels and play an auxiliary role in the treatment of bipolar manic episodes [46]. Bartoli’s study [47, 48] through a meta-analysis on drugs for mania indicated that allopurinol has significant effects on manic symptoms reduction and clinical remission, thus confirming the link between bipolar disorder and uric acid. This study further confirmed that uric acid is involved in the pathogenesis of manic episodes through oxidative stress, and uric acid can be an important biological marker in bipolar manic episodes. As Oliveira [7] found in a ten-year follow-up study of major depressive disorder (MDD) patients, the MDD patients with high uric acid levels have a high risk of turning to bipolar disorder. Serum UA levels showed an excellent accuracy for predicting conversion to BD in inpatients with MDD, high uric acid levels may be can predict the onset of bipolar manic episodes.

In our study, meta-analysis was used to compare the Chinese patients’ differences in uric acid levels between bipolar disorder, healthy controls, depression, and schizophrenia, and analyze the differences between different subtypes of bipolar disorder. However, there are still some problems in our study, such as: ①There are not enough randomized controlled trials in the included studies and there are some heterogeneity in our meta-analysis. ②Since not every study provided specific data such as age and gender, we did not conduct meta-regression analyses, which is a limitation of this study. ③The level of uric acid is easily affected by other factors such as genetics and diet. ④Our findings are not applicable to people other than Chinese population, because our study only included data from Chinese patients. Therefore, to provide references for clinical practice, the association between bipolar disorder and uric acid levels and whether serum uric acid level has diagnostic value for the diagnosis of bipolar disorder still needs more high-quality clinical studies and systematic reviews to confirm.

## Conclusion

Our study showed that Chinese patients with bipolar disorder had elevated UA levels, especially the manic episode in bipolar disorder. In the future, more well-designed studies will be needed to verify the association of serum uric acid levels with bipolar disorder, and whether uric acid levels can be a biomarker for bipolar disorder.

## Data Availability

Derived from the original published studies.
